# Transcriptional Profiling of *ParA* and *ParB* Mutants in Actively Dividing Cells of an Opportunistic Human Pathogen *Pseudomonas aeruginosa*

**DOI:** 10.1371/journal.pone.0087276

**Published:** 2014-01-31

**Authors:** Aneta A. Bartosik, Krzysztof Glabski, Paulina Jecz, Sylwia Mikulska, Anna Fogtman, Marta Koblowska, Grazyna Jagura-Burdzy

**Affiliations:** 1 Department of Microbial Biochemistry, Institute of Biochemistry and Biophysics, Polish Academy of Sciences, Warsaw, Poland; 2 Laboratory of Microarray Analysis, Institute of Biochemistry and Biophysics, Polish Academy of Sciences, Warsaw, Poland; 3 Department of Systems Biology, Faculty of Biology, University of Warsaw, Warsaw, Poland; University of Padova, Medical School, Italy

## Abstract

Accurate chromosome segregation to progeny cells is a fundamental process ensuring proper inheritance of genetic material. In bacteria with simple cell cycle, chromosome segregation follows replication initiation since duplicated *oriC* domains start segregating to opposite halves of the cell soon after they are made. ParA and ParB proteins together with specific DNA sequences are parts of the segregation machinery. ParA and ParB proteins in *Pseudomonas aeruginosa* are important for optimal growth, nucleoid segregation, cell division and motility. Comparative transcriptome analysis of *parA*_null_ and *parB*_null_ mutants versus parental *P. aeruginosa* PAO1161 strain demonstrated global changes in gene expression pattern in logarithmically growing planktonic cultures. The set of genes similarly affected in both mutant strains is designated Par regulon and comprises 536 genes. The Par regulon includes genes controlled by two sigma factors (RpoN and PvdS) as well as known and putative transcriptional regulators. In the absence of Par proteins, a large number of genes from RpoS regulon is induced, reflecting the need for slowing down the cell growth rate and decelerating the metabolic processes. Changes in the expression profiles of genes involved in c-di-GMP turnover point out the role of this effector in such signal transmission. Microarray data for chosen genes were confirmed by RT-qPCR analysis. The promoter regions of selected genes were cloned upstream of the promoter-less *lacZ* gene and analyzed in the heterologous host *E. coli*Δ*lac.* Regulation by ParA and ParB of *P. aeruginosa* was confirmed for some of the tested promoters. Our data demonstrate that ParA and ParB besides their role in accurate chromosome segregation may act as modulators of genes expression. Directly or indirectly, Par proteins are part of the wider regulatory network in *P. aeruginosa* linking the process of chromosome segregation with the cell growth, division and motility.

## Introduction

In eukaryotic cells a defined mitotic apparatus is involved in active segregation of chromosomes to progeny cells during cell division. Studies on numerous low-copy-number plasmids revealed the existence of bacterial counterpart of a mitotic apparatus participating in active partitioning of plasmid molecules to progeny cells, and thereby in their stable maintenance in bacteria [1]. An active plasmid partitioning system consists of two proteins (so called A- and B-type) and an essential *cis*-acting DNA sequence, designated, by analogy to eukaryotic mitotic apparatus, the centromere-like sequence (*parS* or *parC*). The B-type proteins recognize and bind to a specific centromere-like sequence, forming the nucleoprotein complex - segrosome [2]. The A-type proteins are NTPases and provide the dynamic scaffold for segrosome movements. The type of NTPase: Walker-type ATPase, actin-type ATPase and tubulin-type GTPase is the basis for classification of partition systems into three groups: I (variants IA and IB depending on B-component), II and III, respectively [1], [3]. Direct interactions between A and B partners induce the hydrolysis of NTP, which in turn delivers energy for relocation of segrosomes [3], [4].

Recently, many reports have documented the ordered spatial organization of bacterial chromosomes, localization of specific genetic loci to defined regions during the cell cycle, specifically localized replication factories and chromosome segregation controlled in time and space. The majority of existing hypotheses regarding bacterial chromosome segregation are based on active transfer of newly replicated *ori* domains to the poles of the dividing cell. Representatives of ParA (Walker-type ATPases) and ParB (DNA binding proteins with H-T-H motifs) families, homologs of plasmid partitioning proteins from class IA, are postulated as the main players constituting elements of the prokaryotic chromosomal partitioning apparatus [4,5 6,7].

In the majority of chromosomes (except *Enterobacteriaceae* and *Pasteurellaceae*, which are deprived of *par* genes) the genes encoding Par proteins are located in close vicinity of the chromosome replication initiation site - *oriC*, next to the *gid* operon. Together with *rnpA*, *rpmH*, *dnaA*, *recF* and *gyrB* they constitute a conserved cluster of genes whose products play key roles in DNA replication, chromosome segregation and cell division [8], [9]. Highly conserved *parS* sequences have been localized mainly in the so-called *ori* domain of the primary chromosomes (20% of the chromosome around *oriC*). More variability among Par proteins and their centromere-like sequences is observed for the secondary chromosomes of species with more than one chromosome [9].

Studies on *Bacillus subtilis*, *Caulobacter crescentus*, *Streptomyces coelicolor*, *Vibrio cholerae*, *Pseudomonas putida*, *P. aeruginosa*, and, most recently, *Burkholderia cenocepacia*, *Helicobacter pylori*, *Mycobacterium smegmatis*, *Corynebacterium glutamicum* confirmed the participation of chromosomal Par proteins in chromosome segregation to the progeny cells also revealing similarities as well as species-dependent differences. The specific features of the Par proteins in a particular organism are manifested by their involvement in the control of different cellular processes like sporulation, regulation of replication initiation, cell cycle progression, motility or cell-to-cell communication [10], [11], [12], [13], [14], [15], [16], [17], [18].

*P. aeruginosa*, an opportunistic and medically important human pathogen with a simple cell cycle, has become a model for our studies on bacterial chromosome segregation. In the sequenced *P. aeruginosa* reference genome (PAO1 strain - NC_002516) the *parAB* operon is located approximately 7 kb counter clockwise from *oriC* and ten putative *parS* sites for ParB binding have been identified [19]. The closest *parS* sites are located around 4 kb clockwise from *oriC* in the *recF* gene. The *parAparB* operon is transcribed from the weak, *parAp*, located in the upstream *gidB* orf (Lasocki and Jagura-Burdzy, unpublished). The predicted promoter regions of *gidA* and *parA* were cloned in the promoter-probe vector and tested in *E. coli* for the regulation by ParA and/or ParB delivered *in trans* but no regulation was detected (Lasocki and Jagura-Burdzy, unpublished). It cannot be excluded that the nucleoprotein complexes formed at *oriC* and/or *parS*s as well as induced changes in DNA topology might alter the expression of *parAB* genes. Although autoregulation by ParA or ParB protein of *par* operons is well established feature of plasmid partitioning systems [1], [20], [21], [22], in the case of chromosomally encoded Par systems the autoregulation of *par* operons has not been determined.

The *parA parB* genes of *P. aeruginosa* and a single *parS_2_* sequence are able to stabilize otherwise the unstable replicon in *E. coli*, which confirms the partitioning functions of the chromosomal *parABS* system of *P. aeruginosa*. Functional characterization of the Par proteins showed direct ParA-ParB, ParA-ParA and ParB-ParB interactions in the yeast as well as in bacterial two-hybrid system and in *in vitro* studies with purified proteins [16], [19], [23], [24]. *In vivo* experiments in *E. coli* showed that ParB overproduction causes transcriptional silencing of genes in close proximity to *parS_2_* [19]. This feature may play an important role in the folding of the *ori* domain, in regulation of gene expression in this region and in regulation of replication in *P. aeruginosa* [23], [24]. The existence of so-called *ori* domains created by ParB interactions with *parS* sequences was confirmed using *in situ* immunofluorescence. ParB forms a various number (1 to 4) of compact foci on the nucleoid, depending on the stage of the cell cycle and growth conditions [18], [23], [24]. DNA binding activity and polymerization ability of ParB as well as ParA presence determine the distribution and condensation of ParB foci. Our *in vitro* studies have shown that ParA of *P. aeruginosa* exhibits a weak ATPase activity (manuscript in preparation) and is able to bind DNA non-specifically, similarly to ParA homologs from other systems [25]. The pattern of ParA-CFP localization (when plasmid-encoded) in parental strain PAO1161 (WT) and in the *parA*_null_ mutant was dynamic, changing from polar or centrally localized foci to transient haze of fluorescence all over nucleoid. Such ParA patterning disappeared in the *parB*_null_ mutant cells where ParA-CFP signal was seen as dispersed in the boundaries of the nucleoids confirming that ParB was involved in the dynamic behavior of ParA (manuscript in preparation), also featured in other systems [26], [27].

It was shown that overproduction of ParA as well as ParB in *P. aeruginosa* leads to the strong inhibition of bacterial growth [16], [19]. Microscopic observations of cells overproducing ParA and ParB proteins demonstrated disturbances in chromosome partitioning - the effect of DNA guillotining and increased number of extended and chromosome-less cells.

The *parA* and *parB* mutant cultures grown under various conditions showed a slightly extended generation time in comparison with the wild type *P. aeruginosa* grown on a rich medium [16], [18]. Microscopic observations of mutant cells from different phases of culture growth demonstrated a 1000-fold increase in number of the cells with defects in chromosome partitioning. Although these defects were observable in the fast growing cells, they were much stronger under slow bacterial growth conditions in the minimal medium [28]. Defects in chromosome partitioning were accompanied by disturbances in the division cycle (the *par* mutant cells were longer in comparison to the *P. aeruginosa* wild type [24]), by changes in colony morphology as well as defects in swimming and swarming motility, but not in twitching [16], [18]. The ability to perform movements by bacteria of different species is connected with their ability to colonize various ecological niches, and is frequently related to pathogenesis and biofilm formation. The observed impairment of motility of *P. aeruginosa par* mutants suggests direct or indirect role of Par proteins in regulation of these processes.

In this work we focused on the transcriptomic analysis of *parA*_null_ and *parB*_null_ mutants in comparison with parental *P. aeruginosa* PAO1161 strain (here/henceforth WT strain) in order to understand their phenotypes. Comparative transcriptome analysis of cells from logarithmically growing cultures exhibited global changes in gene expression in both analyzed *par* mutants in comparison with the WT strain.

## Results and Discussion

### Mutations in *parA* and *parB* Genes Cause Global Changes in Gene Expression Pattern in *P. aeruginosa*

Previous analysis of PAO1161 *parA*_null_ and *parB*_null_ mutants suggested that ParA and ParB proteins are involved not only in chromosome segregation in *P. aeruginosa* cells, but may also play a broader role connecting chromosome partitioning with chromosome condensation, replication, cell division, regulation of gene expression and controlling different cellular processes in bacteria, e.g. motility and cell-to-cell communication [16], [18].

To determine the changes in gene expression pattern in *parA*_null_ and *parB*_null_ strains of *P. aeruginosa*, microarray analysis was performed. Three biological replicates of each mutant strain including reference WT PAO1161 strain were cultivated in L-broth with OD_600_ measurements and CFU ml^-1^ determination to isolate RNA from logarithmically growing cultures (OD_600_ = 0.5) prior to the microarray analysis.

**Figure 1:**
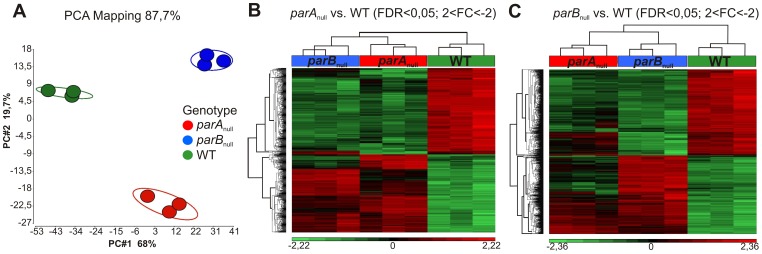
Gene expression analysis in logarithmically growing cultures of *parA*_null_, *parB*_null_ strains versus WT *P. aeruginosa*. (**A**) Quality analysis of three biological replicates of studied strains *parA*_null_, *parB*_null_ and WT of PAO1161 *P. aeruginosa* by principle component analysis (PCA) of data obtained from expression microarray analysis. The first principle component (PC#1) accounted for 68% and the second principle component PC#2 for 19.7% of the total variation in the dataset. The plot indicates that the transcriptome data are of high quality as the samples cluster together according to the strain: green - WT, red - *parA*_null_, blue - *parB*_null_. (**B**) and (**C**) Cluster analysis of the normalized gene expression for genes that were differentially regulated in *parA*_null_ and *parB*_null_ strains as compared to the WT, respectively.

Quality analysis of three biological replicates of studied samples from *parA*_null_, *parB*_null_ and WT strains was done by principal component analysis (PCA) of absolute gene expression. The samples formed clusters for each strain clearly distinct from each other indicating high quality of the transcriptomic data (Figure 1A). In the PCA and hierarchical cluster analysis presented as heat maps in Figure 1B and C, the *parA*_null_ and *parB*_null_ samples are closer to each other than to WT samples, but the results also demonstrate substantial differences between two mutants. Both mutant samples show changes in the transcript profiles in comparison to WT samples as a consequence of inactivation of *parA* and *parB* genes. There is an obvious clustering of identified genes with similar expression patterns in both mutant strains as compared with WT *P. aeruginosa* (Figure 1B and Figure 1C).

**Figure 2:**
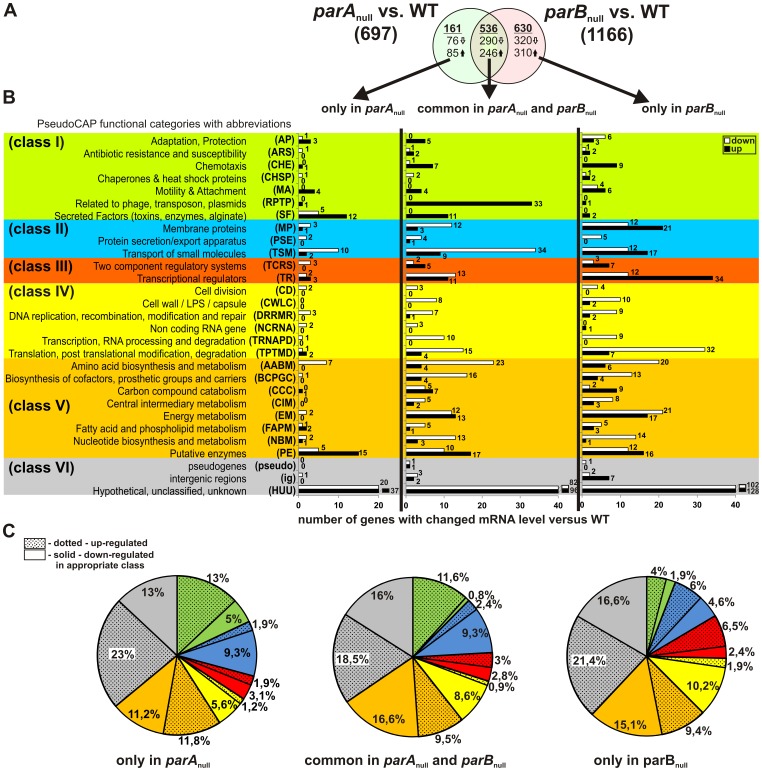
Functional classification of genes differentially expressed in logarithmically growing cultures of *P. aeruginosa par* mutants. (**A**) Venn diagram demonstrating the number of genes with changed mRNA level (fold change ≥2; p-value ≤0.05) in *parA*_null_ and *parB*_null_ mutant strains as compared to reference WT PAO1161 *P. aeruginosa* strain. Three gene set lists were created representing genes differentially expressed only in *parA*_null_, with different expression in both *par* mutants (common in *parA*_null_ and *parB*_null_) and with different mRNA level only in *parB*_null_. (**B**) Functional classification of identified genes according to their predicted or known functions. Functional classes are taken from PseudoCAP [29] and are listed on the left with abbreviations in brackets. The original PseudoCAP functional categories were further grouped into six larger classes encompassing: (**I**) adaptation, protection, motility (green panel); (**II**) membrane proteins, transport, secretion (blue panel); (**III**) signal transduction, regulatory functions (red panel); (**IV**) cellular processes (yellow panel); (**V**) metabolism (orange panel); (**VI**) hypothetical, unknown functions (grey panel). (**C**) The pie charts created for each gene set list illustrating the percentage of genes in each class accounted for the total number of genes with changed expression for: only in *parA*_null_, common in *parA*_null_ and *parB*_null_ and only in *parB*_null_ gene set list.

Comparative transcriptome analysis showed statistically significant (p-value, P≤0.05) over two-fold change in expression levels of 697 loci (331 up-regulated and 366 down-regulated), including six intergenic regions and five tRNA-coding genes for *parA*_null_ samples, and 1166 loci (556 up-regulated and 610 down-regulated), including fourteen intergenic regions and four tRNA-coding genes for *parB*_null_ samples from logarithmically growing cultures of *P. aeruginosa* as compared to WT (Figure 2A and Table 1).

**Table 1:** Number of genes with changed expression in *P. aeruginosa parA*_null_ and *parB*_null_ strains.

*parA*_null_ versus WT						
Change in mRNA level	>2- to 3-fold	>3- to 5-fold	>5- to 10-fold	>10- to 50-fold	>50-fold	Total
Increase	188	75	55	12	1	**331**
Decrease	291	60	12	2	1	**366**
Total	479	135	67	14	1	**697**
***parB*** **_null_ versus WT**						
**Change in mRNA level**	**>2- to 3-fold**	**>3- to 5-fold**	**>5- to 10-fold**	**>10- to 50-fold**	**>50-fold**	**Total**
Increase	248	187	73	39	9	**556**
Decrease	465	128	17	0	0	**610**
Total	713	315	90	39	9	**1166**

The loci with altered expression in *parA*_null_ and *parB*_null_ as compared to reference PAO1161 *P. aeruginosa* (WT), indicated by pairwise comparison of microarray data (fold change FC ≥2; p-value ≤0.05). Number of genes (including intergenic regions and tRNA genes) with indicated mRNA level change are shown. Genes were grouped according to the magnitude of differential expression.

As presented in Table 1 most of the genes with changed mRNA level in mutant samples showed differential expression in the range between 2- to 3-fold: 479 genes from total 697 genes for *parA*_null_ (69%) and 713 genes from total 1166 genes for *parB*_null_ (61%). Among them more than 60% demonstrated a decreased expression. Changes in gene expression of more than 5-fold (up to >50) were observed for 12% of genes in both mutant strains. Interestingly, the genes that exhibited high level of changes in expression are mostly over-expressed in the mutant strains in comparison to the WT strain (82% genes for *parA*_null_ and 88% for *parB*_null_) suggesting the role of Par proteins as the negative regulators.

The complete list of all genes with altered expression in *parA*_null_ and *parB*_null_ in comparison to the WT strain, which exhibited a statistically significant change (fold change, FC ≥2; P≤0.05), is available as Supplementary Information. Two original lists were divided into three gene set lists (according to Venn diagram in Figure 2A), grouping differentially expressed genes in both *par* mutants (Table S1), only in *parA*_null_ (Table S2) or only in *parB*_null_ (Table S3).

Since both mutant strains exhibited similar phenotypes, a substantial amount of overlap in the expression patterns of *parA*_null_ and *parB*_null_ strains had been expected. Indeed, 536 of the 697 genes that had altered expression in *parA*_null_ as compared to WT were also differently regulated in *parB*_null_ (see Venn diagram in Figure 2A). In the set of 536 genes, expression of which was altered in both mutant strains, all genes but one exhibited the same tendency of change (up- or down-regulation) in expression in both mutants. 290 genes were down-regulated and 246 were up-regulated, usually with the higher fold change in the *parB*_null_ mutant strain. Only the PA3365 (*amiB*) gene demonstrated reverse changes in mRNA level when both mutants were compared. It is unclear why PA3365 encoding a probable cytoplasmic chaperone, is 2.1-fold down-regulated in *parA*_null_ and 4.5-fold overexpressed in the *parB*_null_ strain.

It is apparent from the transcriptomic data that lack of ParA and ParB proteins has a great impact on gene expression in *P. aeruginosa.* The 536 genes, indicating expression changes in both mutants and intuitively designated “ParA-ParB regulon”, represent approximately 10% of the PAO1 genome (Table S1). The spectrum of the transcriptomic effects outside of “ParA-ParB regulon” is much broader in the *parB*_null_ mutant strain (630 genes affected) than in the *parA*_null_ mutant strain (161 genes affected) (Table S2 and S3). In either mutant strain the absence of one partner protein promotes the proteolytic cleavage of the second partner [16], [18]. Previous experiments [16] demonstrated that in the total sonicates from *parA*_null_ mutant cells ParB protein was detected in comparable quantities as in the parental strain by Western blotting technique with the use of anti-ParB antibodies (estimated 1000 molecules per cell). However at later stages of the culture growth ParB degradation products were clearly seen and ParB level dropped below detectable (less than 20 molecules per cell). The *parB* insertion had an identical influence on ParA level although no polar effect might have been expected [18]. It was estimated that ParA level dropped from approximately 400 to less than 40 molecules per cell in the *parB* insertion mutant. Since insertional and nonsense mutants in *par* genes behaved similarly ([16] and unpublished) we have decided to use the insertional mutants in *parA* and *parB* genes for the transcriptomic analysis as they facilitated irreversible character of the mutations. Although the transcriptomic analysis revealed that mutation in *parA* gene (insertion of the Sm^R^ cassette) has a polar effect on *parB* expression (*parB* gene exhibits 75-fold decreased expression in *parA*_null_ mutant), the visualization of ParB in *parA*_null_ mutant and the dynamic behaviour of ParA-CFP foci in this mutant (as described in the Introduction) suggest that nevertheless ParB is produced. Both *par* mutants might be considered as deprived of one Par protein and deficient in the second one.

The difference in number of affected genes between the mutants may suggest either the involvement of ParB in many more functions in the cells than ParA or ParA having stronger effect on gene expression when uncontrolled by ParB since we showed that unbalanced production of Par proteins induces severe toxic effects on the cells [16], [19].

### Lack of *ParA* and *ParB* Disturbs Expression of Genes Classified to Different Functional Categories

Transcriptomic analysis of *parA*_null_ and *parB*_null_ mutants when compared to WT *P. aeruginosa* from exponential planktonic culture growth exhibited changes in mRNA levels of genes from all functional categories, according to PseudoCAP function classification of Pseudomonas Genome Database ([29]; www.pseudomonas.com). Figure 2B presents an analysis of differentially expressed genes in *parA*_null_ and *parB*_null_ mutant strains in comparison with the parental strain, based on their PseudoCAP function classification. The diagram in Figure 2B is divided into three columns: genes with altered expression only in *parA*_null_ (left part), only in *parB*_null_ (right part) and a common set of genes exhibiting changes in mRNA level in both *par* mutants (middle part) (in accordance with Table S1–S3). Numerous genes may be classified into more than one functional category according to PseudoCAP, e.g. the PA4218 is assigned to membrane protein (MP), transport of small molecules (TSM) and antibiotic resistance and susceptibility (ARS) functional categories. For simplicity of presentation in Figure 2B, the closely related functional PseudoCAP categories were arbitrarily grouped into six larger classes from I to VI and a single most likely function for each gene was chosen. All assigned PseudoCAP function categories for the identified genes are presented in Tables S1–S3.

Generally, most of the genes grouped in Class I (Figure 2B, green panel), involved in chemotaxis, motility, attachment, adaptation, protection and secretion functions, were significantly induced in the analyzed mutant cells. Genes classified as plasmid and phage related (RPTP) were all up-regulated. The cluster of genes PA0610-PA0648, significantly overexpressed in both *par* mutant cells, encodes proteins involved in pyocin production whose expression is often induced in response to stress conditions. Indeed, a part of the observed changes in gene expression pattern in *par* mutant cells might be the effect of stress response due to the defects in DNA segregation, resulting from incomplete segregation machinery and/or lack of signals coordinating cellular metabolism with chromosome segregation and cell cycle.

Several genes from Class III (Figure 2B, red panel) were significantly induced, especially in *parB*_null_ mutant. Gene products from TR and TCRS categories play an important role in sensing and responding to signals and perturbations within the cell and to environmental stimuli. They are able to modulate and change cellular metabolism to adapt the organism to changing conditions.

In clear contrast to Class I and III, the majority of genes grouped in other classes were down-regulated, as illustrated by pie charts presented in Figure 2C. Most of the identified genes grouped in Class II (Figure 2B, blue panel) were down-regulated in *par* mutants, suggesting impairment of some functions connected with cell membrane.

Genes in Classes IV and V play a crucial role in basic cellular processes and metabolism (Figure 2B, yellow and orange panels). All identified genes involved in cell division, transcription and RNA processing were down-regulated in both *par* mutants (Figure 2B, yellow panel). Essentially, most of the genes from classes IV and V were repressed, suggesting that *parA*_null_ and *parB*_null_ mutant cells had changed their metabolism to slow down basic cellular processes. However, there are a few clusters of genes engaged in nitrogen metabolism and denitrification process (*nir, nar, nap*) that show spectacular overexpression in *par* mutant cells probably as part of a general stress response (see also below).

Class VI (Figure 2B, grey panel), encompassing genes of hypothetical, unknown functions (HUU PseudoCAP category), together with identified pseudogenes and intergenic regions, constitutes approximately 33–35% of all identified genes with altered expression in *par* mutants as compared to WT strain (234 genes of total 697 in *parA*_null_ and 409 genes of total 1166 in *parB*_null_). Expression of 132 genes classified as HUU was enhanced, and expression of 102 genes was repressed in *parA*_null_ strain; in *parB*_null_ mutant the expression of 224 HUU genes was up-regulated and of 185 down-regulated. The common set of HUU genes with changed expression in both *par* mutants includes 96 induced genes and 82 down-regulated ones. The large number of genes in this class exceeded the scale of the diagram and is only schematically presented in Figure 2B.

Comparative transcriptome analysis of *par* mutants cells relative to WT *P. aeruginosa* demonstrated global changes in gene expression pattern in both analyzed mutants. The list of 536 genes with changed expression in both *par* mutants constitutes the most probable candidates for “ParA and ParB regulon” genes and further analysis will mainly focus on this group of genes.

### Lack of *ParA* and *ParB* Affects the Expression of a Number of Transcriptional Regulators

Transcriptional regulators are important factors controlling gene expression and modulating the metabolism and cellular processes as the organism responds to intracellular, as well as environmental signals in order to fulfil its metabolic needs and adapt to the changing growth conditions.

Comparative transcriptomic analysis exhibited a large number of genes encoding known or putative transcriptional regulators or members of two-component regulatory systems with significantly changed mRNA level in *par* mutant strains as compared to WT strain (37 and 80 genes in *parA*_null_ and *parB*_null_, respectively) suggesting an important role of ParA and ParB proteins in the regulatory network of *P. aeruginosa*. The target genes of these regulators might be identified in the present study as being regulated by lack of *parA* and *parB*, although their regulation may be carried via an indirect mechanism. ParA and ParB influenced the expression of a common set of 29 genes (15 activated, 14 repressed) classified as transcriptional regulators (Table 2).

**Table 2:** Transcriptional regulators under control of ParA and ParB proteins.

			*parA*_null_ vs. WT		*parB*_null_ vs. WT		
ID	Gene	Gene product	p-value	FC	p-value	FC	Regulon
**PA0155**	*pcaR*	transcriptional regulator PcaR	0.002	**−2.69**	0.001	**−3.05**	
**PA0167**	*–*	probable transcriptional regulator	0.003	**−2.05**	0.000	**−2.94**	Sress, QS
**PA0236**	*–*	probable transcriptional regulator	0.004	**−2.36**	0.002	**−2.64**	RpoN(KinB), PQS
**PA0479**	*–*	probable transcriptional regulator	0.001	**2.62**	0.000	**5.00**	RpoN(KinB), RpoS
**PA0610**	*prtN*	transcriptional regulator PrtN	0.002	**3.23**	0.002	**3.04**	Stress, QS, RpoN(KinB), PprB
**PA0612**	*ptrB*	repressor PtrB	0.000	**2.98**	0.000	**2.83**	Stress
**PA0797**	*–*	probable transcriptional regulator	0.001	**−3.89**	0.002	**−2.91**	PprB
**PA0961**	*–*	probable cold-shock protein	0.000	**−2.59**	0.001	**−2.53**	
**PA1157**	*–*	probable two-component response regulator	0.000	**−5.20**	0.000	**−5.30**	Stress, PQS
**PA1182**	*–*	probable transcriptional regulator	0.002	**−2.12**	0.001	**−2.28**	
**PA1290**	*–*	probable transcriptional regulator	0.006	**2.53**	0.006	**2.55**	PQS
**PA1431**	*rsaL*	regulatory protein RsaL	0.002	**2.22**	0.001	**2.54**	QS, PprB
**PA1504**	*–*	probable transcriptional regulator	0.000	**−2.58**	0.000	**−2.46**	
**PA2121**	*–*	probable transcriptional regulator	0.000	**3.55**	0.000	**4.79**	RpoS, PQS
**PA2281**	*–*	probable transcriptional regulator	0.000	**−2.19**	0.000	**−2.12**	RpoN(KinB)
**PA2426**	*pvdS*	sigma factor PvdS	0.003	**−2.38**	0.004	**−2.29**	CORE
**PA2449**	*–*	probable transcriptional regulator	0.006	**−2.02**	0.001	**−3.10**	
**PA2572**	*–*	probable two-component response regulator	0.001	**4.57**	0.000	**6.76**	RpoS, QS
**PA2577**	*–*	probable transcriptional regulator	0.001	**2.54**	0.000	**3.41**	RpoS
**PA2622**	*cspD*	cold-shock protein CspD	0.000	**2.39**	0.001	**2.04**	
**PA3027**	*–*	probable transcriptional regulator	0.000	**−2.90**	0.000	**−2.76**	
**PA3458**	*–*	probable transcriptional regulator	0.008	**2.25**	0.000	**9.04**	
**PA3973**	*–*	probable transcriptional regulator	0.013	**5.02**	0.004	**8.34**	RpoS, RpoN(KinB), PprB
**PA4296**	*pprB*	two-component response regulator PprB	0.000	**2.84**	0.000	**6.72**	CORE
**PA4462**	*rpoN*	RNA polymerase sigma-54 factor	0.000	**−2.32**	0.000	**−2.36**	RpoN(KinB)
**PA4781**	*–*	cyclic di-GMP phosphodiesterase	0.000	**4.12**	0.000	**5.01**	RpoS, RpoN(KinB)
**PA4843**	*–*	probable two-component response regulator	0.000	**3.28**	0.000	**4.14**	RpoS
**PA4878**	*–*	probable transcriptional regulator	0.005	**3.63**	0.003	**4.48**	QS
**PA5550**	*glmR*	GlmR transcriptional regulator	0.000	**−2.30**	0.000	**−2.73**	Stress

Genes encoding known or probable transcriptional regulators with changed mRNA level in *P. aeruginosa par* mutants are listed (p-value ≤0.05; fold change FC ≥2). RpoS, QS, PQS, RpoN(KinB), PprB, stress regulated genes are marked (Regulon column) with marked also genes involved in homeostasis maintenance (CORE) according to Table S1.

Among the 15 significantly activated genes encoding transcriptional regulators only a few have been studied in *P. aeruginosa*, e.g. PtrB (PA0612), PrtN (PA0610) involved in stress reaction [30], [31]. For most of them only predicted functions are proposed on the basis of sequence analysis, genomic context and experimental data on homologs in other bacterial species (Table 2). Remarkably, large group of the putative transcriptional regulators induced in the absence of Par proteins (PA0479, PA2121, PA2572, PA2577, PA3973, PA4781, PA4843, PA4878) belong to the RpoS and QS (quorum sensing) regulons [32]. RpoS regulon also contains *pprB* gene significantly induced in both *par* mutants, encoding two-component response regulator PprB. It possesses a CheY-like receiver domain as well as H-T-H DNA-binding domain and is activated due to the phosphorylation by its partner histidine kinase PprA. PprA and PprB regulate the expression of genes that in turn affect membrane permeability and antibiotic sensitivity of *P. aeruginosa* [33], [34]. Comparative transcriptional analysis of *parA*_null_ and *parB*_null_ mutants identified a number of genes PprB-dependent, including several regulatory genes, up-regulated *rsaL, prtN*, PA3973 or down-regulated PA0797 [34]. PA1431 (*rsaL*), up-regulated in *parA*_null_ and *parB*_null_ mutants, encodes the regulatory protein RsaL, involved in negative regulation of QS. RsaL binds simultaneously with LasR to the *rsaL-lasI* bidirectional promoter, thereby preventing the LasR-dependent activation of both genes [35], [36]. Even small changes in the *rsaL* gene expression might have a huge impact on quorum sensing processes dependent on AHL (N-acyl-L-homoserine lactone). Transcription profiling revealed that RsaL regulates 130 genes independently of its effect on QS signal molecule production, including genes involved in virulence [37].

Among genes down-regulated in the absence of Par proteins there are two genes encoding important general transcriptional sigma factors: PvdS and RpoN (Table 2).

PA2426 encodes PvdS sigma factor involved in the expression of pyoverdine biosynthesis genes cluster and genes important for iron uptake and metabolism as well as a number of virulence factors expressed in response to iron starvation [38], [39]. In addition to *pvdS,* other genes engaged in iron uptake were down-regulated in *par* mutant cells, e.g. *fpvA*, pyochelin synthesis genes or *optH*, encoding probable TonB-dependent receptor (Table S1).

**Figure 3:**
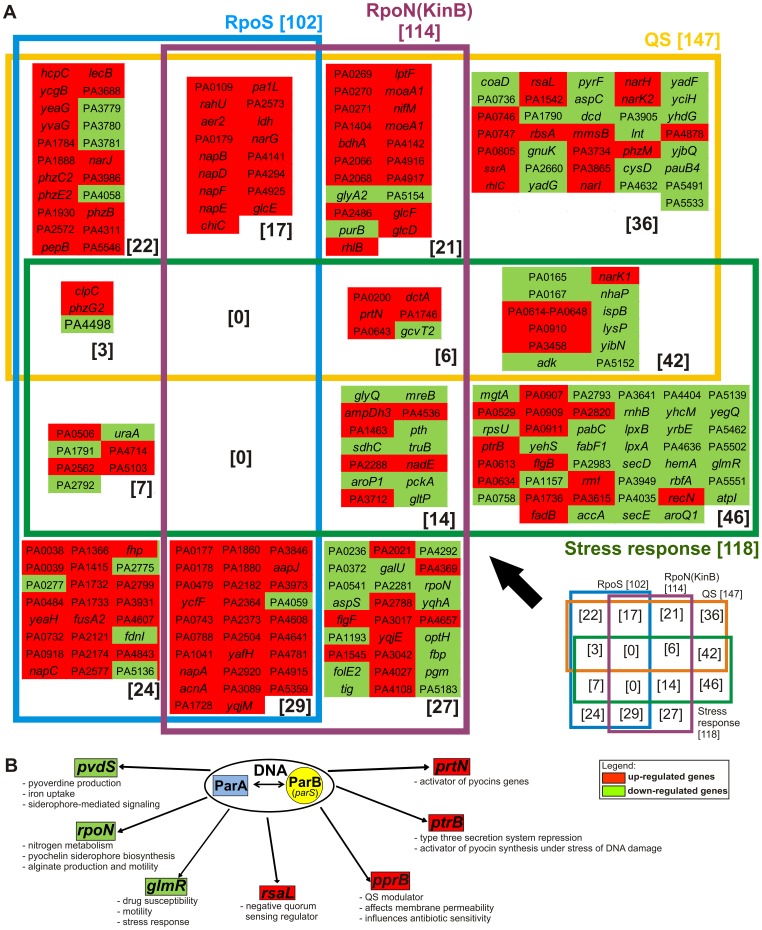
Genes from overrepresented regulons affected by mutations in *parA* and *parB* genes in *P. aeruginosa*. **(A)** RpoS, QS, RpoN(KinB) and stress regulated genes with altered expression in *par* mutants presented as Venn diagram illustrating separate and common genes classified into presented regulons (according to Table S1). (**B)** The most potent known regulators with changed expression in *par* mutants. The functions they influence are given.

PA4462 gene encoding RNA polymerase sigma factor, known as RpoN or sigma factor 54, was approximately 2.3-fold down-regulated in both *par* mutant strains. RpoN controls alginate biosynthesis together with kinase sensor KinB, which phosphorylates AlgB in response to environmental signals. It also controls some regulatory genes and a large number of genes involved in carbohydrate metabolism, quorum sensing, iron regulation, rhamnolipids production, and motility [40], [41]. The expression of a number of genes belonging to the RpoN-KinB regulon was affected in both *par* mutants, indicating also significant overlap between the RpoN-KinB, RpoS and QS regulons, e.g. *nap, nar* or *glc* (Figure 3A).

Among transcriptional regulators repressed in both *par* mutants there is PA0155 gene encoding the PcaR transcriptional regulator. In *P. putida*, PcaR positively regulates the *pca* regulon involved in the chemotactic response to aromatic compounds [42]. PA4974 (*opmH*), a part of the PcaR regulon, encodes probable outer membrane protein precursor, which is also repressed in both *par* mutant strains.

The PA1157 gene expression was approximately 5-fold down-regulated in both *par* mutants. It encodes a putative two-component response regulator, which demonstrates 63% similarity to the transcriptional regulator RstA from *E. coli*. RstA works in pair with the RstB sensor, mainly in stress response cascade in *E. coli* [43].

PA5550 gene encoding GlmR transcriptional regulator was repressed 2–3-fold in both *par* mutant cells. It was demonstrated that mutation in *glmR* caused drug supersusceptibility, loss of motility, reduced resistance to osmotic and heat shock stress, as well as impaired growth at low temperatures, affecting peptidoglycan and LPS synthesis [44].

The predicted effects of changes in production of listed transcriptional regulators seem to lead to the slowing down of metabolism and also to induce the processes related to transition into stationary phase (RpoS regulon). Because a number of RpoS-dependent genes is also QS-dependent (some activated, others repressed at higher cell culture densities), it is not unexpected that QS-dependent communication and regulation of gene expression in *par* mutant cells has been altered.

A short summary of processes that ParA and ParB proteins may influence via control of expression level of known regulatory genes is presented in Figure 3B.

### Lack of *ParA* and *ParB* Alters Expression of Genes Involved in General Stress Response and Maintenance of Cellular Homeostasis

Lack of ParA and ParB causes defects in chromosome partitioning, manifested by appearance of up to 7% of anucleate cells and cells with guillotining chromosomes in *P. aeruginosa par* mutant cultures [16], [18] and that might induce the stress response. In Figure 3 apart from RpoS-, QS- and RpoN(KinB)-dependent genes with changed expression in *par* mutant cells (according to gene lists by Schuster et al. [32] and Damron et al. [40]), the genes involved in stress response are presented (according to Cirz et al. [45]). Among them there are also the *ptrB* and *glmR* genes described above.

Since lack of functional ParA or ParB leads to incomplete chromosome segregation with visible DNA guillotining effects, it was expected that multiple genes from DRRMR functional category (DNA replication, recombination, modification and repair) would be induced. Interestingly, the only gene from this category activated in both *par* mutants was PA4763 (*recN).* It encodes the SOS-inducible DNA repair protein RecN, which in *E. coli* is indispensable for repair of double-strand breaks in the chromosome when these breaks occur at two or more locations [46].

As a part of general stress response in *par* mutant cells, a significant decrease in expression of genes encoding ribosomal proteins, e.g. *rpsU*, *rpsA*, *rpsI*, *prmA* or *rpmH,* was detected (Table S1–S3). Similarly, genes coding for parts of secretion machinery, *secD* and *secE,* were down-regulated in *par* mutant cells as compared with WT *P. aeruginosa*. Secretion protein SecE is encoded in the three-cistronic operon *nusG*-*secE*-PA4276.1. PA4275 (*nusG*) encodes an essential transcription antitermination protein NusG, while PA4276.1 encodes tRNA-Trp. All three genes were repressed in *par* mutants.

In addition, a number of genes with changed expression in both *par* mutant cells is a part of the core set of genes whose products are important for maintaining homeostasis under different stress conditions in *P. aeruginosa* (Table S1; [47]). The set of genes engaged in homeostasis maintenance, significantly repressed in *par* mutants, includes genes encoding for the ferripyoverdine receptor (PA2398), sigma factor PvdS (PA2426), pyochelin (PA4222, PA4223, PA4224, PA4230) as well as the rod shape-determining protein MreC (PA4480), ribose-phosphate pyrophosphokinase (PA4670) and probable xanthine/uracil permease (PA4719).

PA4480 (*mreC*) and PA4481 (*mreB*) genes encoding the rod shape-determining proteins MreC and MreB, were 2–3-fold repressed in analyzed *par* mutants as compared with WT *P. aeruginosa*. In *parB*_null_ also the third gene of the *mreBCD* operon, *mreD*, exhibited a lower mRNA level relative to the WT strain. MreB is an actin homolog forming dynamic helical filaments or patches beneath the surface of the cell, potentially explored as a scaffold for transporting proteins to different locations throughout the bacterial cell. MreB is essential for maintenance of cell shape, chromosome segregation, and polar localization of several bacterial proteins, e.g. type IV pili [48], [49]. The lower level of expression of *mre* operon as well as of a number of genes involved in peptidoglycan synthesis (*mgtA, mltA, htrB, lpxB, lpxA, lnt* and *murA*) in *par* mutant cells may impair their growth.

### Overrepresentation of RpoS- and QS-dependent Genes with Changed Expression in *par* Mutants

Our transcriptomic studies identified many genes from RpoS regulon [32] with altered expression in *par* mutant cells as compared with WT *P. aeruginosa* (Table S1), although the expression of the *rpoS* gene was not changed. Interestingly, among the identified RpoS-dependent genes most showed significant activation in both *par* mutants (Figure 3A). The lack of Par proteins causes visible defects in chromosome segregation in at least 7% of cells in mid-log phase cultures which may explain the slower growth rate [16], [18] and, as demonstrated here, leads to the general stress response and down-regulation of number of genes involved in basic metabolism (class IV and V in Figure 2B) in connection with an earlier induction of RpoS-dependent genes in *par* mutants. RpoS acts as an alternative sigma factor of genes preferentially expressed in stationary phase of growth as well as a regulator of the general stress response.

The list of RpoS-regulated genes with significantly induced expression in *par* mutant cells contains also *orf*s coding for proteins involved in chemotaxis (e.g. PA0176-PA0179, PA1930, PA2573, PA2920, PA4915). Changes in expression level of genes encoding chemotaxis proteins may affect motility of bacteria and explain swimming and swarming defects observed in *par* mutants [16], [18].

Among genes with changed mRNA levels in *P. aeruginosa par* mutant cells there was, in addition to RpoS regulon, a number of genes involved in QS function and control [32], [50]. QS-related genes with changed expression in at least one *par* mutant include those coding for negative regulators of QS: *rsaL*, *rsmA*, *dksA* and *rpoN*, as well as positive regulators like *pprB* [51]. QS-dependent genes encoding phenazine/pyocyanin biosynthesis pathway, rhamnolipids, lectin or chitinase, implicated in pathogenesis, adaptation and survival, were significantly activated (Figure 3).

The group of virulence factors includes also genes encoding proteins involved in motility and coding for flagellar elements (PA1077, PA1081) as well as, mentioned above, chemotaxis sensory transducer (PA2573) or two-component response regulator (PA2572), playing a role in the first stages of infection, mainly during adhesion to host cells. Similarly, PA4108, PA4781 and PA2572 are classified as coding virulence factors due to their involvement in regulation of adhesion and biofilm formation by modulation of c-di-GMP level. All these genes were induced in one or both of the analyzed *par* mutant strains.

One of the metabolic processes regulated by QS is regeneration of the AHL precursors such as methionine and S-adenosylmethionine (SAM) and degradation of adenosine via inosine and hypoxanthine [52]. A number of genes whose products are involved in AHL metabolism, are down-regulated in both or at least one of the analyzed *par* mutant strains, e.g. PA0390 (*metX*), PA0430, PA0654 (*speD*), PA1687 (*speE*), PA3169 (Table S1–S3).

Among genes under RpoS and QS control with changed mRNA level in both *par* mutants, the high activation was observed for the cluster of *nar* genes (10–40-fold induction) and *nap* genes (4-10-fold up-regulation). Seven *nar* genes (PA3871- PA3877) are organized in an operon encoding respiratory nitrate reductase components. The *nar* operon is activated under low-oxygen tension conditions and in the presence of nitrate, and transcriptional regulators Anr, NarXL and Dnr [53].

The PA1172-PA1176 *nap* genes code for cytochrome c-type, an essential component of the electron transport chain, participating in periplasmic nitrate reduction. In concert with activation of *nar, nap* genes, the expression of *nirS* (coding for nitrite reductase precursor), *nirQ* (encoding regulatory protein NirQ with MoxR-like ATPase motif), *norCBD* (encoding cytochrome c subunits of nitric oxide reductase and denitrification protein NorD) were induced in *parB*_null_ mutant. The highest activation in *par* mutants was exhibited by the PA2664 gene (41-fold in *parA*_null_ and 296-fold in *parB*_null_). PA2664 encodes flavohemoprotein with a role in detoxification of NO (nitric oxide) under aerobic conditions [54]. NO is the intermediate but also an important effector of denitrification pathway. It activates transcriptional regulator Dnr (Dissimilative Nitrate Respiration regulator), acting jointly with NarXL and Anr for activation of *nar*, *nir*, *nor* and *nos* operons. However *dnr* was 4.2-fold repressed in *parA*_null_, but not affected in *parB*_null_. Interestingly, the genes encoding elements of two-component regulatory system the *narLX*, were also differently expressed in *par* mutants. The *narX* gene was 2-fold down-regulated in *parA*_null_ relatively to WT, while the *narL* gene was 2.9-fold activated in *parB*_null_ mutant. It is a peculiar discrepancy since they usually co-activate the *nar* genes, but it is possible that such situation takes place when Par proteins are present in the cell. It suggests that the effect of the denitrification pathway induction may be achieved by different means in two *par* mutants.

It is unclear why there is such a high overexpression of *nar, nap* and *nif* genes in cultures of exponentially growing *par* mutants cells where oxygen concentration and nutrients availability should not yet be limiting factors for cells to grow and divide. Most likely, it can be explained by activation of the denitrification process as a part of the stress-response cascade in reaction to the cellular signals mimicking the necessity of cells to turn to the less-active metabolically state, characteristic for stationary phase cultures where lack of oxygen is a real problem. The denitrification processes in *P. aeruginosa* are controlled not only by low-oxygen tension but also by the cell-to-cell communication signals [55]. In *P. aeruginosa* two chemically distinct signaling molecules have been characterized: AHLs (produced by LasI and RhlI) and PQS (Pseudomonas Quinolone Signal: 2-heptyl-3-hydroxy-4-quinolone, produced by *pqs* operon). The AHL-dependent regulators LasR (through RhlR) and RhlR repress the denitrification operons so it is likely that derepression of the denitrification pathway is the result of the decrease in AHLs synthesis (see above). The second effector PQS acts posttranslationally inhibiting activities of NAR, NOR and NOS reductases, but stimulating NIR (NO_2_^-^ reductase) that produces NO. High amounts of NO induce genes important for NO detoxification e.g. PA2664 *fhp* (activated in both mutants to the extreme levels), PA2665 encoding transcriptional activator FhpR and PA2663 involved in activation of the production of polysaccharides virulence-related factors, such as pyoverdine, PQS, elastase [56], at the same time reducing swimming and swarming motility (both genes are over-expressed in the *parB* mutant). NO at non-toxic levels regulates the social behaviour through regulation of level of another secondary messenger c-di-GMP by inducing enzymes involved in its degradation (see next section).

### Lack of *ParA* and *ParB* Induces Expression of Genes Involved in c-di-GMP Turnover and Signalling

Cyclic-di-GMP (cyclic diguanylate) is an important messenger ubiquitous in bacterial cells controlling various processes, e.g. switch between the motile planktonic and biofilm lifestyles of bacteria, virulence of animal and plant pathogens, antibiotic production, progression through the cell cycle and other cellular functions [57]. The level of c-di-GMP in the cell is tightly controlled by the opposite actions of two classes of enzymes. Proteins encoding HD-GYP or EAL domains are specific phosphodiesterases (PDEs) involved in hydrolysis of c-di-GMP, whereas diguanylate cyclases (DGCs) possessing the GGDEF domain are engaged in the production of c-di-GMP from two GTP molecules [57]. The GGDEF, EAL and HD-GYP domains are usually linked to various N-terminal sensory input domains, often transmembrane, suggesting that numerous environmental and cellular signals are integrated into the c-di-GMP signalling network. Cyclic-di-GMP is bound by transcriptional regulators, proteins containing PilZ domains, proteins carrying degenerate GGDEF or EAL domains, as well as RNA in riboswitches modulating protein-protein interactions, protein-DNA interactions, protein enzymatic activity, protein-RNA interactions and by transcription, translation and other cellular processes [58].

Among the genes involved in c-di-GMP turnover with altered expression in at least one *par* mutant, three genes were found encoding diguanylate cyclases (DGCs) with GGDEF domain (PA0169, PA2771, PA4843), five genes encoding proteins with both GGDEF and EAL domains (PA0861, PA2567, PA3311, PA4367, PA5017) and three genes coding for phosphodiesterases with HD-GYP motif (PA2572, PA4108, PA4781). For some genes, RpoS-dependent transcription was suggested (Table S1; [32]).

Significant overexpression (2-7-fold) of three genes coding for phosphodiesterases may cause a decrease in c-di-GMP level in *par* mutants cells promoting a higher expression of flagellar components and virulence factors genes. Indeed, we observed overexpression of a number of genes encoding flagellar proteins and this overproduction could lead to motility dysfunction. Some genes encoding virulence factors also seemed to be overexpressed in *par* mutant strains, e.g. *lecA, lasA, lasB, hcpC, apr, rhl* and *phz.*

The PA4843 gene up-regulated 3-4-fold in both *par* mutant strains encodes a protein classified into TCRS containing response regulator with a CheY-like receiver domain important in sensing signals from the environment and a GGDEF domain. It shows 50% similarity to *pleD* gene product responding to c-di-GMP level required for the swarmer-to-stalked-cell transition in *C. crescentus* [59].

Because so many genes whose products are involved in c-di-GMP turnover exhibited spectacular changes in expression in *par* mutant cells (especially in the *parB*_null_ mutant) it was particularly interesting to find the effectors of modulated level of c-di-GMP action in *P. aeruginosa*. Recent studies of Duvel et al. [60] described the proteomic method allowing for identification of c-di-GMP binding proteins in *P. aeruginosa*. By comparing the list of genes encoding proteins identified in c-di-GMP pull-down experiments [60] with the list of genes whose expression was affected by mutations in *parA* and *parB*, we found 25 genes in *parA*_null_, 54 in the *parB*_null_ mutant, and common 20 genes in both mutant strains, whose activity might be regulated by c-di-GMP. Among those with significantly up-regulated mRNA level in both *par* mutant cells were genes encoding: PA4781 c-di-GMP phosphodiesterase (described above); PA0176 aerotaxis transducer Aer2; PA2573, PA2920, PA4915 and PA2788, predicted membrane chemotaxis transducers; PA2799 and PA4608, hypothetical proteins possessing the PilZ domain (a known receptor for the second messenger c-di-GMP, with homology to type IV pilus assembly protein); PA3458, probable transcriptional regulator. These few examples show that the spectrum of c-di-GMP effectors is broad and the impact of altered expression of some c-di-GMP efector proteins from one side and differences in expression of genes encoding DGC and PDE from another side in *par* mutant cells may trigger a large cascade of effects/defects. Some of them seem to be connected with chemotaxis, motility, signal transduction and regulatory functions, but for others the role in *P. aeruginosa* regulatory network and metabolism is waiting to be elucidated.

### Validation of Microarray Results by RT-qPCR

RT-qPCR (reverse transcription followed by quantitative PCR) was performed to confirm the observed changes in gene expression in *parA*_null_ and *parB*_null_ strains as compared with WT. The same RNA samples were used in RT-qPCR analysis as those used in microarray analysis. In the first step RNA from three biological replicates of each strain was used in reverse transcription reaction to obtain cDNA. For selected genes specific primers were designed (Table S4) and used in qPCR with cDNA as a template.

Genes listed in Table 3 were chosen for RT-qPCR analysis because their fold change in expression in *par* mutants varied significantly across a relatively broad range and some of them were physiologically relevant candidates for further analysis. The constitutively expressed housekeeping gene *nadB* (PA0761), which encodes an L-aspartate oxidase and expression of which was not altered in *par* mutants, was used as an internal control in qPCR reactions. Essentially all the RT-qPCR results correlated with the microarray alterations (promotion or repression) in gene expression. PA2398 (*fpvA*), PA4307 (*pctC*), PA4675 (*optH*) and PA5139 showed reduced expression, while PA0586, PA1081, PA1930, PA2570, PA2572, PA2573, PA2920, PA3520, PA3688, PA3973, PA4108 and PA4843 demonstrated increased expression in both *par* mutants as compared with WT (Table 3). For the PA1196 and PA2567 genes, mild overexpression in *parA*_null_ mutant (<1.8-fold) was detected using the qPCR method that was not included in the microarrays data with cut-off of fold change >2. Using RT-qPCR analysis significant overexpression of PA3006 gene was detected in both *par* mutants relatively to the WT strain whereas microarray analysis indicated changes only in the *parB* mutant. On the basis of RT-qPCR analysis, the PA3006 gene is also considered as a part of the ParA/ParB regulon.

**Table 3:** Validation of microarray data by RT-qPCR analysis.

			*parA*_null_ versus WT				*parB*_null_ versus WT			
			fold change				fold change			
ID	Gene	Product	MC	RT	SD RT	change	MC	RT	SD RT	change
**PA0586**	*ycgB*	conserved hypothetical protein	5.81	**5.85**	0.43	up	6.36	**4.32**	0.16	up
**PA1081**	*flgF*	flagellar basal-body rod protein FlgF	3.90	**4.70**	0.11	up	3.26	**2.59**	0.47	up
**PA1196**	*–*	probable transcriptional regulator	nd	**1.37**	0.19	up	7.25	**7.59**	0.46	up
**PA1930**	*–*	probable chemotaxis transducer	6.60	**2.86**	0.80	up	17.53	**3.47**	1.61	up
**PA2398**	*fpvA*	ferripyoverdine receptor	−4.26	**−1.51**	0.01	down	−8.31	**−8.10**	0.42	down
**PA2567**	*–*	cyclic di-GMP phosphodiesterase class I	nd	**1.73**	0.26	up	5.38	**4.44**	0.90	up
**PA2570**	*pa1L*	PA-I galactophilic lectin	6.95	**6.14**	1.06	up	8.26	**6.53**	1.19	up
**PA2572**	*–*	probable two-component response regulator	4.57	**3.93**	0.37	up	6.76	**3.27**	0.04	up
**PA2573**	*–*	probable chemotaxis sensory transducer	4.37	**6.75**	0.57	up	7.36	**6.51**	1.23	up
**PA2920**	*–*	probable chemotaxis transducer	2.49	**4.55**	0.95	up	9.39	**8.01**	1.23	up
**PA3006**	*psrA*	transcriptional regulator PsrA	nd	**5.23**	0.14	up	7.80	**5.78**	0.60	up
**PA3520**	*–*	–	14.82	**8.04**	1.50	up	21.12	**7.74**	1.65	up
**PA3688**	*–*	–	7.39	**5.68**	0.34	up	9.48	**2.61**	0.35	up
**PA3973**	*–*	probable transcriptional regulator	5.02	**5.78**	0.13	up	8.34	**6.22**	0.39	up
**PA4108**	*–*	cyclic di-GMP phosphodiesterase class II	3.63	**3.78**	0.10	up	6.93	**4.39**	0.94	up
**PA4307**	*pctC*	chemotactic transducer PctC	−5.78	**−3.61**	0.25	down	−3.83	**−4.86**	0.57	down
**PA4675**	*optH*	probable TonB-dependent receptor	−5.08	**−2.94**	1.32	down	−3.58	**−4.67**	0.69	down
**PA4843**	*–*	probable two-component response regulator	3.28	**3.79**	0.46	up	4.14	**3.88**	0.47	up
**PA5139**	*–*	–	−5.08	**−2.43**	0.45	down	−5.27	**−5.65**	0.23	down

The RT-qPCR on RNA samples applied for microarrays analysis for chosen genes with changed mRNA level in *P. aeruginosa par* mutants (p-value ≤0.05; fold change ≥2). Abbreviations: MC - microarray data; RT - RT-qPCR data; SD RT - standard deviation for RT-qPCR analysis; nd - change not detected. Standard deviation from at least three independent experiments is presented.

### *parA* and *parB* act as repressors and activators of gene expression

**Figure 4:**
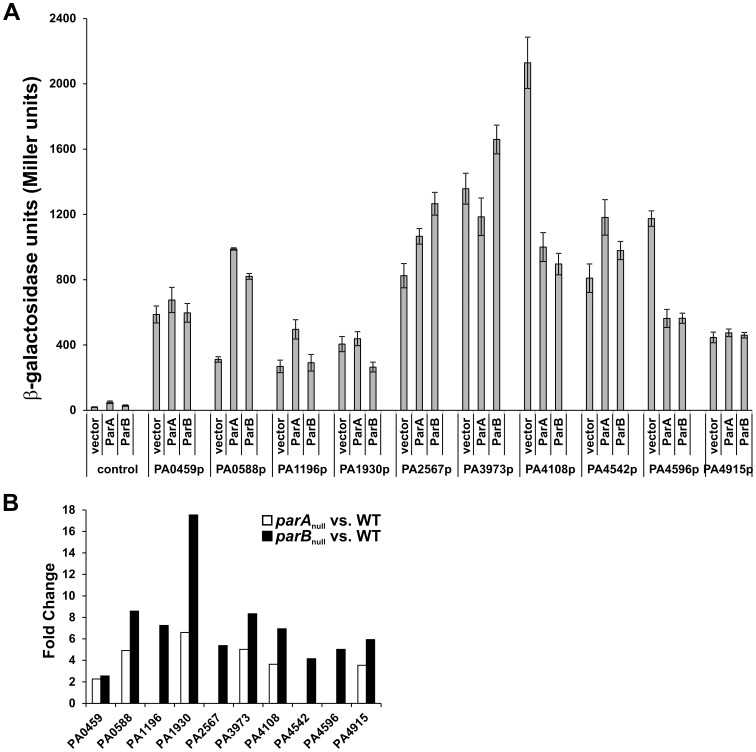
Regulation of gene expression by ParA and ParB of *P. aeruginosa*. **(A)** The β-galactosidase activity in *E. coli* DH5Δ*lac* transformants bearing pCM132 derivatives with analyzed promoter regions-*lacZ* fusions and pGBT30 derivatives [72] expressing ParA, ParB or no protein as a control (vector). **(B)** Fold changes determined by comparative microarray analysis of *P. aeruginosa parA*_null_, *parB*_null_ versus WT strains (p-value ≤0.05; fold-change ≥2) for chosen genes which promoter regions were analyzed using promoter-*lacZ* fusions in (A).

Ten putative promoter regions of PA0459, PA0588, PA1196, PA1930, PA2567, PA3973, PA4108, PA4542, PA4596 and PA4915 genes, selected on the basis of transcriptomic data, were amplified by PCR and cloned into the broad host-range promoter-probe vector pCM132 [61]. The expression of the transcriptional fusions of studied promoter with *lacZ* was analyzed in transformants of three *E. coli* strains, namely: DH5Δ*lac* (pGBT30), DH5Δ*lac* (pKLB1 *tacp-parA*) and DH5Δ*lac* (pKLB2 *tacp-parB*) in overnight cultures without induction of *tacp* (Figure 4A). All cloned regions contained promoter sequences, that led expression of the reporter *lacZ* gene in *E. coli* DH5Δ*lac* (pGBT30) strain. The lowest β-galactosidase activity was detected for *PA0588p*, *PA1196p*, *PA1930p*, *PA4915p* fusions (less than 500 U), the highest activity was observed for *PA4108p-lacZ* fusion (above 2000 U).

When the activities of the promoters were tested in the presence of ParA or ParB produced from the compatible high-copy-number plasmid, the level of *lacZ* expression was clearly affected for most of the tested sequences (Figure 4A). Although the *par* genes were inserted under control of *tacp*, no IPTG was used to further induce the expression in order to avoid the possible “toxic” effect of overproduced proteins. Our previous studies confirmed production of the appropriate protein detected by Western blot analysis at the basal level expressed from the *tacp* promoter without IPTG induction in transformant cultures (data not shown).

The direct regulation of *lacZ* expression by Par proteins complying with the transcriptomic data was shown for three out of ten tested promoters *PA4108p*, *PA4596p* and *PA1930p* (Figure 4). Two promoters were not affected by ParA and ParB when tested in the heterologous host (*PA0459p* and *PA4915p*).

Interestingly, in clear contrast with transcriptomic data and observed induction in *P. aeruginosa par* mutants, the expression of *PA0588p-, PA1196p-, PA2567p-, PA3973p-* and *PA4542p*-*lacZ* fusions revealed stimulatory effect of ParA, ParB or both proteins under tested conditions (Figure 4). The lack of regulation or reverse regulatory effect of Par proteins on *P. aeruginosa* promoters in the *E. coli* may indicate the complex regulation of expression of the studied promoters in their host. Indeed, expression of some of them depends on the potent *P. aeruginosa* regulators: RpoS, RpoN or PprB (*PA0588p*, *PA2567p*, *PA3973p*) [32], [40].

Performed regulatory experiments in *E. coli* for promoter regions of chosen *P. aeruginosa* genes in the presence of ParA and ParB demonstrated that both proteins are able to modulate gene expression acting directly or indirectly as repressors or activators. Further studies are needed to explain obtained results in the context of complex regulatory network influencing gene expression in *P. aeruginosa.*

### Conclusions

The role of ParA and ParB in chromosome segregation and organization in *P. aeruginosa* has already been documented [16], [18], [19], [23], [24], [28], but their influence on gene expression has not yet been studied. This is the first report considering ParA and ParB proteins as regulators of gene expression. The *parA*_null_ and *parB*_null_ mutations do not lead to the lethality [16], [18] although they disturb the proper segregation of the newly replicated genomes. The populations of mutants which produce up to 7% of anucleate cells and many more cells with aberrantly segregated chromosomes grow on a rich medium with only slightly prolonged division time (36 min for *parA*_null_, 33 min for *parB*_null_ versus 30 min for WT) [16], [18], [24], [28]. Phenotypic characterization of *P. aeruginosa parA*_null_ and *parB*_null_ mutants exhibited defects in swarming and swimming motility [16], [18]. These defects might be correlated with altered expression of genes involved in motility, chemotaxis, or signal transduction functions (Table S1–S3; Figure 2B, 3, 4). Additionally, genes encoding products involved in c-di-GMP signalling [57] might influence motility functions in *par* mutant cells, e.g. PA2567, PA4108. The *par* mutant cells are impaired in motility and form colonies with altered morphology. The question arose how the cells might cope with disturbances in chromosome segregation and survive as the population.

The comparative transcriptome analysis of *parA*_null_, *parB*_null_ mutant populations from the mid-log phase of growth of planktonic cultures versus the parental PAO1161 strain of *P. aeruginosa* demonstrated global changes in gene expression pattern. Despite the good nutrients and oxygen supply, genes of the RpoS regulon were mostly induced suggesting entry of bacteria into a less metabolically active state. Delaying the cell growth and division might provide the bacteria with a chance to segregate the chromosomes by mechanisms alternative to *par* system. Functional categorization of identified genes demonstrated an increase in expression of genes whose products are engaged in adaptation, protection and motility function with clear down-regulation of genes that encode proteins involved in basic metabolism and cellular processes (Figure 2). In addition, a number of genes encoding parts of two-component regulatory systems and transcriptional regulators exhibited changes in mRNA level in *par* mutant cells.

The intriguing question is the signal prompting such delay and RpoS regulon activation. Our transcriptomic studies point out the importance of two signalling molecules: NO, the intermediate and effector of denitrification pathway, which regulates the cell-to-cell signalling, here induced as the result of stress response and c-di-GMP involved also in quorum sensing. The levels of both molecules might be affected in *par* mutants since the expression of genes involved in their synthesis and decay is significantly altered. There is another signalling molecule, PQS, which is considered to be an essential mediator of the survival strategies for bacterial population and one of these strategies is to enter a dormant state that slows down metabolism. The 38 genes dependent on PQS show an altered expression in ParA-ParB regulon (Table S1), however, the mechanism of PQS action is still not fully understood. PQS is postulated to act not only as the transcriptional regulator, but also as the posttranscriptional modulator [62]. Further studies may identify the signal responsible for slowing down the metabolism in mid-log phase of culture growth and inducing adaptive responses.

Another open question is the mode of action of Par proteins as the potential regulators in the WT *P. aeruginosa*. *In vivo* regulatory experiments showed direct regulation of some genes by Par proteins, playing the role of repressors (*PA4108p, PA4596p, PA1930p*) as well as activators (*PA0588p, PA2567p, PA4542p, PA1196p, PA3973p*) (Figure 4A). The altered expression of a large number of genes encoding known or predicted transcriptional regulators in *parA*_null_ and *parB*_null_ mutants suggests also the indirect regulation mediated by regulatory genes under ParA and ParB control.

It has been postulated for the chromosomal homologs that ATP bound-ParA interacts non-specifically with DNA and ParB by stimulating ATPase activity releases ParA from the nucleoid [26], [27], [63]. In the absence of ParB, ParA is most probably in its dimer state bound with ATP proficient to bind non-specifically DNA. One of the reasons of the global changes in genes expression in *P. aeruginosa parB*_null_ mutant might be the effect of uncontrolled action of ParA on DNA in the absence of ParB. On the other hand ParB interacts with *parS* sequences and is able to spread on flanking DNA regions possibly influencing gene expression [18], [19]. Creation of large nucleoprotein complexes greatly influences DNA topology and this might be an additional level of control of genes expression.

A hierarchical cascade of direct and indirect regulation by ParA and ParB may be enhanced by signal molecules mentioned above. Among them, the secondary messenger c-di-GMP emerges as an important factor. The c-di-GMP signalling is involved in the regulation of change of the lifestyle from planktonic to biofilm or controlling the virulence determinants in bacteria [57]. Recently, Baraquet et al. [64] demonstrated that FleQ/FleN/DNA interactions are modulated by c-di-GMP, changing the mode of action of FleQ from repressor to activator, which in turn influenced exopolysaccharide production. Moreover, the cell cycle dependent fluctuations of c-di-GMP were visualized in bacteria, showing the asymmetrical distribution of c-di-GMP correlated with the time of cell division and polar localization [65]. The local concentration of c-di-GMP might be crucial for macromolecular complexes allowing independent and parallel control of different output reactions.

**Figure 5:**
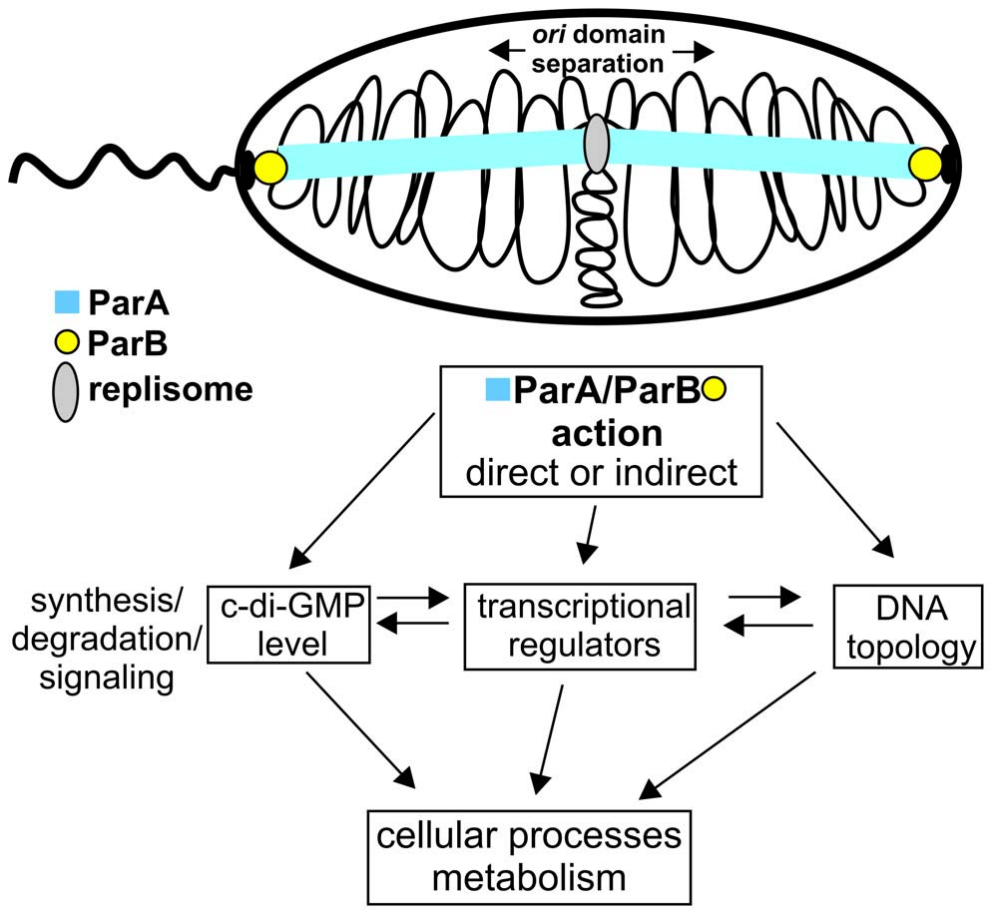
Model of ParA and ParB action in *P. aeruginosa*. The chromosome is illustrated as thin black line, the replisome as grey ellipse, ParA structures as overlapping blue squares, ParB as yellow circle, cell envelope and single polar flagella as thick black line (see text for description).

On the basis of performed studies and present knowledge we propose the model of ParA and ParB action in *P. aeruginosa* cells (Figure 5). The ParA and ParB proteins play a major role in chromosome segregation in bacterial cells. ParB interacts with *parS* sequences and helps to organize, condense and orient the newly replicated *oriC* regions. ParB interactions with ParA stimulate ParA ATPase activity and redistribution of the large nucleoprotein complexes to opposite halves of the cell prior to cell division. An additional role of ParA and ParB action on DNA is modulation of gene expression with an opportunity to coordinate different processes within the bacterial cell cycle including chromosome condensation, organization, segregation, chromosome replication, cell division and growth rate. The mode of action of ParA and ParB is complex. It may involve direct interactions with promoter regions of certain genes, as well as it might be the consequence of specific (*parS* sequences) and unspecific interactions with DNA and induction of topological changes. All these mechanisms may influence expression of specific targets or regulatory genes with a potential to regulate subsequent genes, as a part of the regulatory network. ParA and ParB interactions with partner proteins may also modulate the process (unpublished data). The molecular mechanisms explaining the mode of ParA and ParB action in gene regulation need further investigations. This study provides useful information about the possible links between chromosome segregation, the progression of the cell cycle, control of the growth rate by intertwining with RpoS regulon, QS-regulatory networks and regulation of genes expression.

## Materials and Methods

### Bacterial Strains and Growth Conditions

*Pseudomonas aeruginosa* PAO1161 (*leu^-^ r* ^-^), derivative of PAO1, was kindly provided by B. M. Holloway (Monash University, Clayton, Victoria, Australia). *P. aeruginosa* PAO1161 Rif^R^ (WT), PAO1161 Rif^R^ *parA1-39::smh* (*parA*_null_) and PAO1161 Rif^R^ *parB1-18::Tc^R^* (*parB*_null_) strains were obtained as described previously [16], [18], [71]. *E. coli* K12 strain DH5α was used for standard cloning procedures whereas *E. coli* DH5Δ*lac* {Nal^r^; *deoR thi1 relA1 supE*44 *endA*1 *gyrA*96 *recA*1 *hsdR*17 Δ(*argF lac*) U169} was used for regulatory studies.

Bacteria were grown in L broth [66] at 37°C or on L agar (L-broth with 1.5%, w/v, agar) supplemented with antibiotics as appropriate: 10 µg ml^-1^ for chloramphenicol resistance and 50 µg ml^-1^ for kanamycin resistance in *E. coli.*

Cells of *P. aeruginosa* PAO1161 strains were taken from a deep-frozen stock, spread on L-agar plates and grown overnight at 37^o^C. Bacteria from single colonies were then used to inoculate L-broth liquid cultures and grown overnight with shaking at 37^o^C. Three independent overnight cultures for each strain were diluted 1∶100 in fresh L-broth and propagated with shaking at 37^o^C. Samples were collected from the cultures at regular intervals to measure the optical density at 600 nm (OD_600_) and to determine the CFU/ml. Aliquots of 4 ml for exponential phase culture (OD_600_ = 0.5; in total 2×10^9^ cells) were subjected to RNA extraction.

### RNA Isolation

Three independent replicates of total RNA were isolated from each strain using an RNeasy mini-kit with on-column DNase digestion (Qiagen) according to the manufacturer’s instructions. The DNase digestion using TURBO DNase kit (Ambion) was used to eliminate DNA contamination. The RNA quality and integrity was checked using Bioanalyzer (Agilent Technologies) and the concentration was estimated using Nano Drop ND-1000 Spectrophotometer.

### Affymetrix Genechip Microarrays

For DNA microarrays, three biological replicates of total RNA (10 µg) from each strain were used for cDNA synthesis, fragmentation, and labeling according to the Affymetrix GeneChip Expression Analysis Technical Manual (Affymetrix, Santa Clara, CA, USA). Briefly, random hexamer primers (final concentration, 25 ng µl^-1^; Invitrogen) were added to the total RNA (10 µg) along with *in vitro* synthesized *B. subtilis* control spikes. cDNA was synthesized using Superscript II (final concentration of 25 U µl^-1^ (Invitrogen) according to the manufacturer’s instructions under the following conditions: 25°C for 10 min, 37°C for 60 min, 42°C for 60 min, and 70°C for 10 min. RNA was removed by alkaline treatment and subsequent neutralization. cDNA was purified using a MiniElute PCR Purification Kit (Qiagen) and was eluted in 12 µl of EB Buffer. cDNA was fragmented with DNase I (0.6 U per µg of cDNA; Amersham) at 37°C for 10 min and then end-labeled with biotin-ddUTP using a GeneChip® DNA Labeling Reagent (Affymetrix) at 37°C for 60 min. Fragmented labeled cDNA samples were hybridized to the array (GeneChip *P. aeruginosa* Genome Array) and scanned with Affymetrix GeneChip Scanner 3000.

### Microarray Data Analysis

Microarray gene expression data were analyzed using Partek Genomic Suite 6.6 beta software (Partek Inc., St. Louis, MO). Raw data (.cel files) were imported and processed using GeneChip Robust Multiarray Averaging (GC RMA) background correction, quantile normalization, Log2 transformation and median polish summarization. Principal component analysis (PCA) was used to check the batch effect and to identify the outliers. Analysis of variance (ANOVA) using REML (restricted maximum likelihood) was performed in order to identify differentially expressed genes for a particular genotype versus wild type (*parA*_null_ vs. WT and *parB*_null_ vs. WT). Gene lists were created using a cut off of p-value with FDR (False Discovery Rate) ≤0.05, with a fold change 2 (−2≥ FC ≥2). Hierarchical clustering of significantly and differentially expressed genes was performed to group samples with similar expression patterns into clusters.

Microarray as well as data analysis were performed in the Laboratory of Microarray Analysis, Department of Systems Biology, Warsaw University and Institute of Biochemistry and Biophysics PAS, Warsaw, Poland (www.corelab.pl).

### RT-qPCR Analysis

The same RNA samples were used for RT-qPCR analysis in order to verify microarray data for chosen genes. Three biological replicates of total RNA (2 µg) from each strain served as a template for cDNA synthesis with SuperScipt VILO Master Mix reverse transcriptase (Invitrogen). The cDNA was purified using QiaQuick PCR purification Kit (Qiagen) and then used as a template in qPCR performed with SYBR® Green JumpStart™ *Taq* ReadyMix kit (Sigma). Three biological replicates with three technical replicates per each were used for each gene. The specific qPCR primers, were used to amplify reference and target genes. Sequences of primers used for RT-qPCR analysis are available in Table S4. Before use, primers were tested for equal efficiency of the qPCR reactions. The efficiency of the quantitative PCR reaction with each primer pair was calculated and used to calculate the ratio of each studied gene to the reference gene. Only efficiency values of about 0.95 or more were accepted. For each cDNA sample, three reactions were carried out using two template amounts of 20–60 ng, each in duplicate. The quality of results was evaluated based on expected Ct differences between the two cDNA amounts as well as product melting curves. Changes in individual gene expression between the WT and mutant strain were calculated with normalization of Ct values to mean Ct value for *nadB* (PA0761) reference housekeeping gene using the Pfaffl method [67], [68]. RT-qPCR analysis using *P. aeruginosa* housekeeping gene *proC* (PA0393) as an internal normalizer confirmed no changes in expression of *nadB* gene (ratio 1) in *parA*_null_, *parB*_null_ versus WT strains of *P. aeruginosa* (data not shown).

qPCR was performed using the Light Cycler 480 (Roche). PCR products were detected with SYBR green fluorescent dye and amplified according to the following protocol: one cycle at 95°C for 5 min, followed by 45 cycles at 95°C for 15 s and 60°C for 1 min. The melting curve was 65 to 95°C with increments of 0.5°C/s. Each PCR mixture contained the following: 5 µl SYBR Green JumpStart Taq ReadyMix for quantitative PCR (Sigma), 2 µl of diluted cDNA, and each of the forward and reverse primers at 0.4 µM; nuclease-free water was added to obtain a final volume of 10 µl. In each run, negative controls (no cDNA) for each primer set were included.

### Regulatory Experiments with Promoter-*lacZ* Fusions and *parA* or *parB* Expressed *in trans* in *E. coli*

The putative promoter regions of chosen genes were PCR amplified on the genomic DNA of the PAO1161 as the template using appropriate pairs of primers listed in Table S4. Amplified regions after EcoRI-BamHI digestion were inserted into the broad-host-range pCM132 promoter probe vector after EcoRI-BglII cleavage upstream to the promoter-less *lacZ* reporter gene (see Table S5). The empty vector pCM132 with the deletion of EcoRI-BglII fragment (as a negative control) and its derivatives with inserted promoter regions were transformed into competent cells of *E. coli* DH5Δ*lac* (pGBT30), DH5Δ*lac* (pKLB1 *tacp-parA*) and DH5Δ*lac* (pKLB2 *tacp-parB*) strains. Competent cells of *E. coli* were prepared by the standard CaCl_2_ method [69]. The β-galactosidase activity in liquid overnight cultures of transformants was analyzed as previously described [70]. Three independent assays for at least three independent transformants in one set of experiment were performed. The average of three independent experiments is presented.

### Microarray Data Accession Number

The raw microarray data supporting the results of this article were deposited in the NCBI‘s Gene Expression Omnibus (GEO) database (http://www.ncbi.nlm.nih.gov/geo/) and is accessible through GEO Series accession number GSE47031 (release after publication acceptance).

## Supporting Information

Table S1 **The** ***P. aeruginosa*** **ParAB regulon genes.** The list of genes differentially expressed in *parA*_null_ and *parB*_null_ (common in *parA*_null_ and *parB*_null_ list) as compared with reference PAO1161 *P. aeruginosa,* indicated by pairwise comparison of microarray data (fold change FC ≥2; p-value ≤0.05). All assigned PseudoCAP function categories [29] for the identified genes are presented as abbreviations (see legend). RpoS, QS, PQS, RpoN(KinB), PprB, stress regulated genes are marked (Regulons column) with marked also genes involved in homeostasis maintenance (CORE in Regulons column), according to appropriate references [32], [50], [62], [40], [34], [45], [47]. The last column indicates genes from Venn diagram presented in Figure 3A with genes set marked by appropriate number in bracket.
(XLS)

Table S2 **Genes differentially expressed only in** ***parA*** **_null_ mutant.** The list of genes differentially expressed only in *parA*_null_ mutant versus reference WT PAO1161 strain (fold change FC ≥2; p-value ≤0.05), not present on the list for *parB*_null_ mutant microarray data. All assigned PseudoCAP function categories [29] for the identified genes are presented as abbreviations (see legend).
(XLS)

Table S3 **Genes differentially expressed only in** ***parB*** **_null_ mutant.** The list of genes differentially expressed only in *parB*_null_ mutant versus reference WT PAO1161 strain (fold change FC ≥2; p-value ≤0.05), not present on the list for *parA*_null_ mutant. All assigned PseudoCAP function categories [29] for the identified genes are presented as abbreviations (see legend).
(XLS)

Table S4 **Primers used in this work.**
(DOCX)

Table S5 **Plasmids used in this work.**
(DOCX)
