# Supplementary material for: Transcriptional Profiling of ParA and ParB Mutants in Actively Dividing Cells of an Opportunistic Human Pathogen Pseudomonas aeruginosa
Source: PLoS One. 2014 Jan 31;9(1):e87276. doi: 10.1371/journal.pone.0087276 (PMC3909081; doi:10.1371/journal.pone.0087276)
Supplement: Table S4 — Primers used in this work. (DOCX) [file pone.0087276.s004.docx]

**Table S4. Primers used during this work.**

| **Abb. Nr** | **Name** | **Sequence 5´-3´** |
| --- | --- | --- |
| Primers used in qPCR analysis | | |
| #1 | **NADBF** | CTACCTGGACATCAGCCACA |
| #2 | **NADBR** | GGTAATGTCGATGCCGAAGT |
| #3 | **PA0586F** | CCTGTTCCGCACCTGGACCG |
| #4 | **PA0586R** | CCGTAGCGCTCCTCGCACTG |
| #5 | **PA1081F** | CAGCAACGTGAACGCGGTGG |
| #6 | **PA1081R** | CCGAATCGTCCTCGGCGGTG |
| #7 | **PA1196F** | TGGAAAGTACCCTGTTCGGC |
| #8 | **PA1196R** | ATCTCGTCGAGGAACAAGCTG |
| #9 | **PA1930F** | GGGCAGAACGTCGCCACCAT |
| #10 | **PA1930R** | ACCAGCTCGTCCGCCGAGTA |
| #11 | **PA2398F** | CGACCAACCCCGCCATCACC |
| #12 | **PA2398R** | CCAGCCTGAACCTGCCAGCC |
| #13 | **PA2567F** | CTCGGTCTGCTGGAAGAAGG |
| #14 | **PA2567R** | GATGGAAATGAGCGCGATGG |
| #15 | **PA2570F** | TATCTACAATCCGGGCGATG |
| #16 | **PA2570R** | GACCAGCGCACCACAAAA |
| #17 | **PA2572F** | GAGGAGCTAAAGCGCAGCTA |
| #18 | **PA2572R** | CTGGTTGGTCTGCTTGTCCT |
| #19 | **PA2573F** | CCGCGTCGGTGGTCGATGAG |
| #20 | **PA2573R** | GCGAACGCTGGGCGATGAGT |
| #21 | **PA2920F** | GGATCGGGTGCGCCAGTACG |
| #22 | **PA2920R** | CGCGCTCCAGAGGGCATAGC |
| #23 | **PA3006F** | CCTGTCGATCTTCATGCGCT |
| #24 | **PA3006R** | ACCTTGCCGTAGACCTCCTC |
| #25 | **PA3520F** | ATCCTGCACGGCGTCGATCC |
| #26 | **PA3520R** | ACGCCTCGAGGAATGCGTGC |
| #27 | **PA3688F** | CGCATGGCCCGCAACCAGTA |
| #28 | **PA3688R** | GCTGCGCCGCGGATACATCT |
| #29 | **PA3973F** | GGATCCTGAAGTCGACGAGC |
| #30 | **PA3973R** | GAAAGCTGGAATGCGCCAC |
| #31 | **PA4108F** | GCCACGGGTCAAGGTATTCT |
| #32 | **PA4108R** | GACGATTCGGTCGGTCTGTC |
| #33 | **PA4307F** | AACAGCCCGTCTTCAGCCGC |
| #34 | **PA4307R** | AACCTTCCGGCATGGCGTCG |
| #35 | **PA4675F** | TCAGCGTGAACGACTCGCCG |
| #36 | **PA4675R** | AGAACGCCGCCACCTGGGTA |
| #37 | **PA4843F** | CTTCGCCGAGATCCACTACC |
| #38 | **PA4843R** | TTGATATCGAGGTCCGGGGT |
| #39 | **PA5139F** | TTTTCCACCGCCCGTACGCC |
| #40 | **PA5139R** | GCCGCGAGCCATCAGTACCC |
| #41 | **PROCF** | CAGGCCGGGCAGTTGCTGTC |
| #42 | **PROCR** | GGTCAGGCGCGAGGCTGTCT |
| Primers used in standard PCR reactions | | |
| #43 | **pPA0459F** | GCGAATTCCATGGCCCAGGAACTTTG |
| #44 | **pPA0459R** | GCGGATCCTGAAGTATTCGGCGATGT |
| #45 | **pPA0588F** | CGGAGCTCGATCGCCTTTCTTCCTTTACTCCG |
| #46 | **pPA0588R** | GCGGATCCTAACGGTCACAGCCGATGG |
| #47 | **pPA1196F** | GCGAATTCGTGCGAGCGATGATGTG |
| #48 | **pPA1196R** | GCGGATCCTCCCGGATGTCGAAATCT |
| #49 | **pPA1930F** | CGGAGCTCGATCGAAGTGGGTTTCGGGATACG |
| #50 | **pPA1930R** | GCGGATCCGCGCACGACTCCTTGATG |
| #51 | **pPA2567F** | GCGAATTCACATGGCGTCCTGGTAAG |
| #52 | **pPA2567R** | GCGGATCCATCGGCAATCAATGATGTC |
| #53 | **pPA3973F** | GCGAATTCGACGGCGTACTGCTCGAC |
| #54 | **pPA3973R** | GCGGATCCTCACGCTTCAGGCTTTGC |
| #55 | **pPA4108F** | GCGAATTCACATGCTTCTTCTCCACAGT |
| #56 | **pPA4108R** | GCGGATCCATTGCGTATCGCTTTGTATC |
| #57 | **pPA4542F** | GCGAATTCATGGCCGACATCTA |
| #58 | **pPA4542R** | GCGGATCCGTAGATAAGGGCGATTTTCC |
| #59 | **pPA4596F** | GCGAATTCGTCCTGTCAAAGGCGTTC |
| #60 | **pPA4596R** | GCGGATCCGTCGAGACTGTCCCTGTG |
| #61 | **pPA4915F** | GCGAATTCATCGAGTGGATGGTAGAGC |
| #62 | **pPA4915R** | GCGGATCCGAGCATCGTCAGCGGTAG |
