# Supplementary material for: Transcriptional Profiling of ParA and ParB Mutants in Actively Dividing Cells of an Opportunistic Human Pathogen Pseudomonas aeruginosa
Source: PLoS One. 2014 Jan 31;9(1):e87276. doi: 10.1371/journal.pone.0087276 (PMC3909081; doi:10.1371/journal.pone.0087276)
Supplement: Table S5 — Plasmids used in this work. (DOCX) [file pone.0087276.s005.docx]

**Table S5. Plasmids used during this work.**

| Plasmid | Relevant features | Reference/Source |
| --- | --- | --- |
| **pCM132** | *ori*_ColE1_, *oriV_IncP_, oriT, traJ’, trfA*, Km^R^, promoter-less *lacZ* | [61] |
| **pGBT30** | *ori*_MB1_, Ap^R^, *lacI*^Q^ *tacp*, expression vector | [72] |
| **pKLB1** | pGBT30 with *tacp*-*parA* transcriptional fusion | [19] |
| **pKLB2** | pGBT30 with *tacp*-*parB* transcriptional fusion | [19] |
| **Plasmids constructed during this work.** | | |
| **pCAB132** | modified pCM132 with deletion of EcoRI-BglII fragment | |
| **pGJB459** | pCM132 derivative with cloned *PA0459p-lacZ* fusion | |
| **pGJB588** | pCM132 derivative with cloned *PA0588p-lacZ* fusion | |
| **pGJB196** | pCM132 derivative with cloned *PA1196p-lacZ* fusion | |
| **pGJB930** | pCM132 derivative with cloned *PA1930p-lacZ* fusion | |
| **pGJB567** | pCM132 derivative with cloned *PA2567p-lacZ* fusion | |
| **pGJB973** | pCM132 derivative with cloned *PA3973p-lacZ* fusion | |
| **pGJB108** | pCM132 derivative with cloned *PA4108p-lacZ* fusion | |
| **pGJB542** | pCM132 derivative with cloned *PA4542p-lacZ* fusion | |
| **pGJB596** | pCM132 derivative with cloned *PA4596p-lacZ* fusion | |
| **pGJB915** | pCM132 derivative with cloned *PA4915p-lacZ* fusion | |
